# Supplementary material for: Treatment Outcomes and Significance of Multimodal Treatment in Esophageal Squamous Cell Carcinoma with Synchronous Oligometastasis
Source: Cancers (Basel). 2025 Oct 23;17(21):3407. doi: 10.3390/cancers17213407 (PMC12609970; doi:10.3390/cancers17213407)
Supplement: Supplementary file 1 [file cancers-17-03407-s001.zip › cancers-3913672-supplementary.pdf]

Supplementary Material

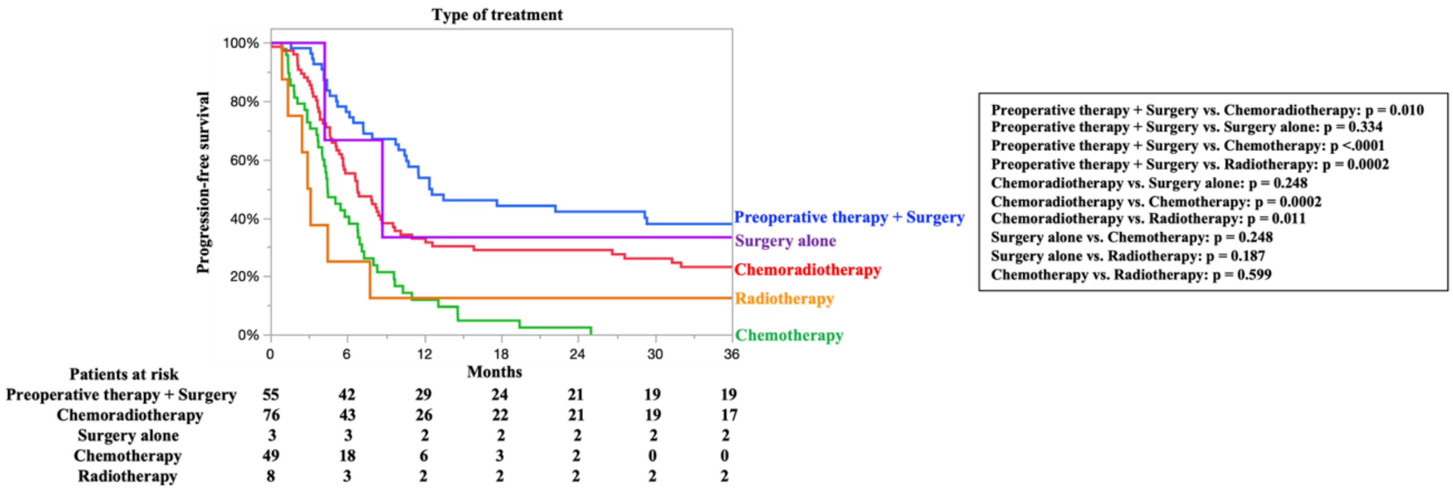

Figure S1. Progression-free survival according to treatment type

Patients in the chemotherapy group were included in the immunotherapy group. The 3-year PFS rates for preoperative therapy plus surgery, chemoradiotherapy, chemotherapy, surgery alone, and radiotherapy were 37.9, 23.1, 0, 33.3, and 12.5%, respectively. The median PFS for preoperative therapy plus surgery, chemoradiotherapy, chemotherapy, surgery alone, and radiotherapy was 12.4, 6.8, 4.4, 8.7, and 3.0 months, respectively. Patients who underwent preoperative therapy plus surgery and chemoradiotherapy had significantly better OS than those who underwent other treatments.

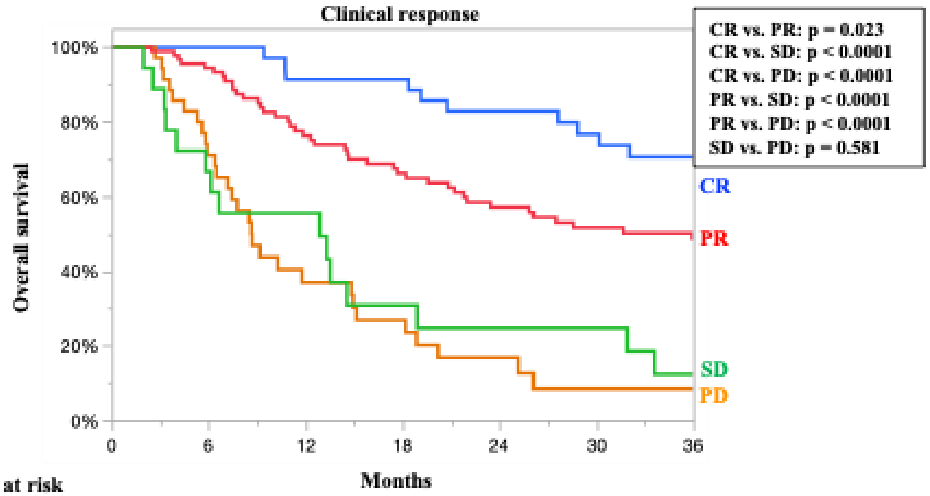

Patients at risk							
CR	35	35	33	33	29	26	24
PR	93	82	62	53	43	38	33
SD	18	13	10	6	5	5	3
PD	36	25	12	9	5	3	2

Figure S2. Overall survival (OS) according to clinical response

OS according to clinical response is shown in Fig. 2b. Three cases treated with surgery alone and six cases with no assessment were excluded. The 3-year OS rates for complete response (CR), partial response (PR), stable disease (SD), and progressive disease (PD) were 70.5, 48.6, 12.3, and 8.4%, respectively. The median OS time for CR, PR, SD, and PD were 92.4, 35.9, 12.8, and 8.6, respectively. Patients with CR had a significantly better OS than those with PR, SD, or PD (p=0.023, p<.0001, p<.0001). Patients with PR had a significantly better OS than those with SD or PD (p<.0001, p<.0001).
